# An Emotion Recognition Method for Humanoid Robot Body Movements Based on a PSO-BP-RMSProp Neural Network

**DOI:** 10.3390/s24227227

**Published:** 2024-11-12

**Authors:** Wa Gao, Tanfeng Jiang, Wanli Zhai, Fusheng Zha

**Affiliations:** 1 Co-Innovation Center of Efficient Processing and Utilization of Forest Resources, Nanjing Forestry University, Nanjing 210037, China; 2 College of Furnishings and Industrial Design, Nanjing Forestry University, Nanjing 210037, China; 3 The State Key Laboratory of Robotics and System, Harbin Institute of Technology, Harbin 150001, China

**Keywords:** emotion recognition, robot’s emotional body movements, neural network, human–robot interaction

## Abstract

This paper mainly explores the computational model that connects a robot’s emotional body movements with human emotion to propose an emotion recognition method for humanoid robot body movements. There is sparse research directly carried out from this perspective to recognize robot bodily expression. A robot’s body movements are designed by imitating human emotional body movements. Subjective questionnaires and statistical methods are used to analyze the characteristics of a user’s perceptions and select appropriate designs. An emotional body movement recognition model using a BP neural network (EBMR-BP model) is proposed, in which the selected robot’s body movements and corresponding emotions are used as inputs and outputs. The corresponding topological architecture, encoding rules, and training process are illustrated in detail. Then, the PSO method and the RMSProp algorithm are introduced to optimize the EBMR-BP method, and the PSO-BP-RMSProp model is developed. Through experiments and comparisons for emotion recognition of a robot’s body movements, the feasibility and effectiveness of the EBMR-BP model, with a recognition rate of 66.67%, and the PSO-BP-RMSProp model, with a recognition rate of 88.89%, are verified. This indicates that the proposed method can be used for emotion recognition of a robot’s body movements, and optimization can improve emotion recognition. The contributions are beneficial for emotional interaction design in HRI.

## 1. Introduction

Emotional interaction in human–robot interaction (HRI) has become an important issue, as humanoid robots are widely applied in different kinds of scenarios like education [1,2], healthcare [3,4,5], shopping [6,7], exhibitions [8], etc. Robots should be capable of communicating emotionally with users. It can make a user’s robot easy to understand and promote interaction in a positive direction. The emotional interaction in HRI can be achieved through facial expressions, voice, body movements, etc. For example, many studies have explored emotional interaction from the perspective of facial expressions, like the impact of affective facial expressions in robots on human behaviors and attitudes [9,10], the estimation and recognition of a user’s emotion [11,12], the generation of robot facial expressions [13,14], etc. There are also many research works referring to affective voices, such as in the evaluation of different robot voices [15,16,17,18], emotional expressions of synthesized speech [19,20,21], etc. Lots of studies focused on combinations of different approaches, such as facial expressions and vocal information, voice and head movements, and so on [22,23,24,25]. However, compared with these mentioned works, there have still been limited studies in the field of robot body movements with emotions up to now, especially for the corresponding emotion recognition.

As body language plays an important role in communicating human emotions in social interactions [25], emotional body movements also have irreplaceable advantages for HRI. They can provide a feasible approach, perceive emotions from a long distance, help identify emotional information when voices are not clear, and also benefit emotion recognition when facial expressions are ambiguous. There have been studies to verify that the recognition accuracy of the affective states expressed by body movements is comparable relative to facial expression [26,27]. Our research focuses on the emotion recognition of a humanoid robot’s body movements during HRI. We hope to explore the relationship between a humanoid robot’s body movements and its emotional state and develop a computational model to recognize the emotions of a humanoid robot’s body movements, which will make the future human-like emotional expression systems of robots in HRI more convenient and natural.

To this end, the design of emotional body movements of robots and the user’s perception of emotions expressed through a robot must be considered. To date, there have been studies working on the design of emotional body movements of robots from different points of view, and the robot platforms Nao, Brian 2.0, Pepper, Teleco, etc., are employed and designed to express different emotions such as joy, anger, shame, sadness, warmth, and so on [28,29,30,31]. However, just like how human body movements are constrained by bones and joints, the implementation of body movements into robots is also constrained by their physical structure. The robot platform we used, a Yanshee robot, has a relatively lower degree of freedom (DoF) compared with the robots mentioned above, which makes it difficult to verify the transferability of body movements designed in other studies. Hence, the design of emotional body movements in this paper is carried out by imitating human emotional body movements, and then they are analyzed to verify the corresponding emotional category.

As mentioned above, to develop the emotion recognition method for a humanoid robot’s body movements during HRI, the work in this paper is described in detail as follows:

1. Construct an effective dataset consisting of the robot’s emotional body movements and corresponding emotional states and explore the trends of user perceptions for different emotions.
2. Propose a computational model to build the mapping between the robot’s emotional body movements and corresponding emotional states and achieve emotion recognition of the robot’s body movements.
3. Verify the effectiveness of the proposed model with experiments on emotion recognition for the emotions of a robot’s body movements and find a feasible approach to optimizing the recognition rate.

**Figure 1:**
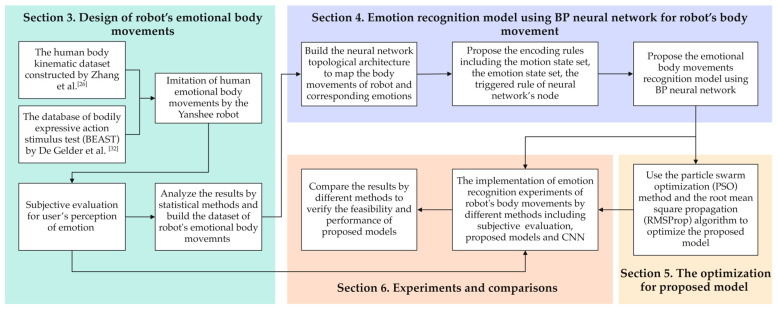
The block scheme for the main procedure of this study [26,32].

The structure of this paper is as follows. Section 2 briefly illustrates the related works, including the design of a robot’s emotional body movements and the corresponding perceptions of users in HRI, as well as the neural network used for emotion recognition in HRI. Section 3 shows the designed robot’s emotional body movements and the implemented procedures and analysis for subjective evaluation. Section 4 proposes an emotion recognition model using a BP neural network for the robot’s body movement. Section 5 illustrates the optimization for the proposed model using PSO and RMSProp. Section 6 illustrates the experiments on emotion recognition through different methods and describes their comparisons. Section 7 shows the conclusions and gives some limitations of this study. The main research procedure of this study is shown in Figure 1. The relationship between Section 3, Section 4, Section 5 and Section 6 is clearly illustrated to improve readability.

## 2. Related Works

### 2.1. Design of Robot Emotional Body Movements and Corresponding Perceptions of Users in HRI

Designing a robot’s body movements to accurately express emotions is quite important in the field of robotics. At present, three approaches for this purpose can be summarized. The first is to consider the relationships between the characteristics of body movements and emotions, which can be used to classify different emotional types. For instance, positive and negative emotions can be distinguished by trunk movements, ranging from stretching to bowing [33]; vertical features and features indicating the lateral opening of the body are informative for separating happiness from sadness [34]; and happy and angry expressions usually accompany movements such as bouncing and foot stomping, respectively [35]. Such features have been used in some studies for the design of emotional body movements for robots. Tsiourti et al. employed a Pepper robot and designed body movements reflecting happiness with a straight robot trunk, head bent back, vertical and lateral extension of the arms, and large, fast movements; body movements reflecting surprise with a straight trunk, a backward step, and fast movements; and body movements reflecting sadness with a bowed trunk, downward head, and slow body movements [28]. Fu et al. employed the mobile CommU robot and designed gait-induced vertical oscillations simulating overall body movements to express the emotions of happiness, sadness, and anger [36]. The second approach is to imitate human emotional movements. Robots can express emotions by imitating the motions of users [24,37,38]; for instance, Erden employed a Nao robot and generated the emotional body movements associated with anger, disgust, fear, happiness, sadness, and surprise based on the simplified human body models presented by Coulson [29,39]. The third approach involves designing robot emotional behaviors using motion primitives. Humans can accomplish different tasks by assembling motion primitives (MPs) [40]. Some studies have introduced MPs to express robot motions such as anthropomorphic arms or emotional body movements [40,41,42]. The works of Wei et al. and Li at al. employed Nao robots and provided proof that adopting MPs to combine robot movements with emotional expression in HRI is possible [40,41].

To assess the user’s perception of the emotional body movements of a robot, subjective and objective evaluations can be considered. Subjective evaluation is the main approach for evaluating the bodily emotional expressions of robots, generally adopting self-reported methods such as questionnaires, interviews, and so on. Users are asked to watch the body movements of the robot, then choose and rate the emotions they feel according to pre-determined categories. In the studies of Tsiourti et al. [28], Erden [29], Fu et al. [36], Wei et al. [40], Li et al. [41], Urakami [43], and others, different questionnaires were used to evaluate the perceptions of robot emotional body movements from multiple views, such as emotion understandability, anthropomorphism, likeability, and so on. Objective evaluations involve obtaining user emotional responses through physiological measures, such as eye-tracking, electroencephalogram (EEG), electrocardiogram (ECG), electrodermal activity (EDA), and so on [44,45]. Related studies focused on objective evaluation in HRI have been increasing in recent years. For example, Guo et al. studied the influence of a humanoid robot’s emotional behaviors on the emotional responses of users through subjective reporting, eye-tracking, and EEG [46]. Staffa et al. collected the EEG signals of 10 participants demonstrating either a positive or negative personality who interacted with a Pepper robot, and demonstrated the possibility of classifying a user’s emotional responses to the robot’s behavior from the EEG signals [47].

### 2.2. The Neural Network Used for Emotion Recognition in HRI

The aim of the computational model to be built in this study is to construct a mapping between the robot’s emotional body movements and corresponding emotional states. The use of a neural network is a feasible approach for this purpose, due to their non-linear, self-organization, and self-adaptation characteristics, among others. Neural networks are often used for emotion recognition in different contexts [48,49,50]. The design for emotional body movements of robots and the corresponding perceptions of user emotions can be used as the inputs and outputs, respectively, for the computational model to be developed. To date, multiple neural networks have been adopted for emotion recognition via facial expression, body gesture, and speech in the HRI context [51,52,53,54], but those focused on emotion recognition with respect to robot body movements remain rare. In the work of Li et al., an artificial emotion expression method that could recognize the emotion patterns expressed by a robot according to its motion behaviors was proposed, for which the generalized regression neural network (GRNN) and the discrete octagonal Pleasure–Arousal–Dominance (PAD) model were used [41]. Furthermore, some studies in the field of facial expression recognition in HRI have focused on the relationships between facial actions and emotions, which can serve as a reference, to some extent. For example, Rawal et al. introduced the expression generation network (ExGenNets), which can reconstruct simplified facial images from robot joint configurations with a classifier network for facial expression recognition [55]. Khashman proposed an emotional back propagation (BP) neural network with two emotional parameters—anxiety and confidence—for facial recognition [56]. Li et al. proposed an emotion recognition system for a humanoid robot based on a convolutional neural network (CNN) and six basic emotions—happiness, anger, disgust, fear, sadness, and surprise—and used a long short-term memory (LSTM) recurrent neural network to build the relationship between the transformation of facial expressions and the six basic emotions [57]. Li et al. built a facial expression and touch gesture emotion dataset and proposed a multi-eigenspace-based multimodal fusion network (MMFN) to recognition tactile–visual bimodal emotion in the HRI context [58]. The results of these studies demonstrate that neural networks can be used to construct the relationship between robot motions and human emotion, providing a feasible approach to develop the corresponding computational model.

## 3. Design of Robot’s Emotional Body Movements

### 3.1. Materials

**Figure 2:**
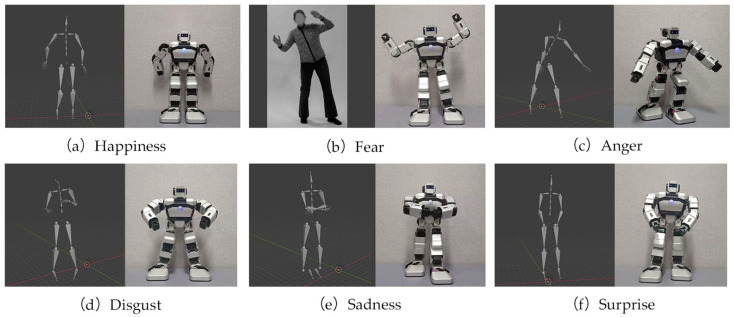
The emotional body movements of human from the works of Zhang et al. and De Gelder et al., respectively, and the corresponding imitations by Yanshee robot. The behaviors shown from (**a**–**f**) are with emotions happiness, fear, anger, disgust, sadness, and surprise, respectively.

A humanoid robot named Yanshee was employed to imitate the emotional body movements of human. The Yanshee robot utilizes the open hardware platform architecture of Raspberry Pi + STM32, and consists of 17 DoFs. The body movements that the Yanshee robot imitates come from two databases: a human body kinematic dataset constructed by Zhang et al. and a database for the bodily expressive action stimulus test (BEAST) constructed by De Gelder et al. In the work of Zhang et al., 22 semi-professional actors provided a total of 1402 recordings of body movements, with happiness, sadness, anger, fear, disgust, surprise, and neutral emotional expressions [26]. The database constructed by De Gelder et al. is composed of 254 whole-body expressions from 46 actors expressing emotions, including happiness, sadness, anger, and fear [32]. A total of 72 body movements reflecting 6 emotions—including happiness, fear, anger, disgust, sadness, and surprise—are mimicked by Yanshee (i.e., 12 for each emotion), with 32 of the body movements being imitated from the work of Zhang et al., and 40 of them from the work of De Gelder et al. The videos of these body movements were labelled from V1 to V72. Examples of the robot’s body movements imitating different emotions are shown in Figure 2. In Figure 2a–f, the videos V10, V8, V4, V12, V2, and V6 correspond to the pre-determined emotions of happiness, fear, anger, disgust, sadness, and surprise, respectively.

The behaviors of the humans and robots in Figure 2 visually illustrate the imitation of human emotional behaviors by the Yanshee robot. When imitating, some difficulties were found due to the limited DOFs of Yanshee. For example, the contrast between human and Yanshee robot poses are quite clear in Figure 2d. The Yanshee robot cannot achieve the sloping shoulder posture, as it does not possess the associated activity structures. Although it makes little difference while posing, the emotional perception of the user may change [59].

### 3.2. Subjective Evaluation for User’s Perception of Emotion

**Figure 3:**
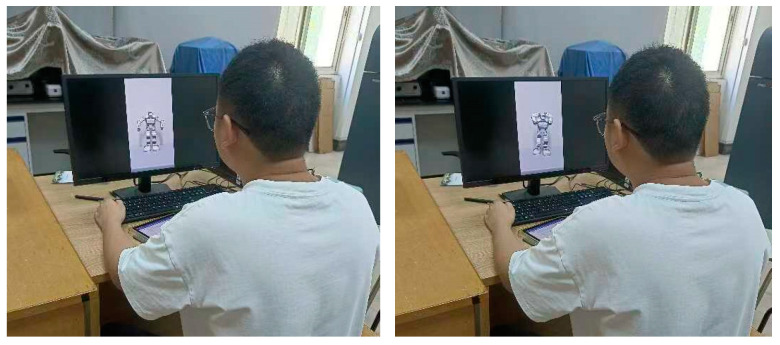
Scenario of a student watching video V41.

Considering that robots are limited by their physical structure, imitating human body movements does not necessarily make users feel the same emotion as that in the datasets. Hence, we need to verify whether the emotions identified belong to the pre-determined emotional categories. A total of 51 students with age ranging from 20 to 27 years (M = 23.55, SD = 1.39) were recruited from the same university, including 26 males and 24 females. All of them had normal or corrected-to-normal vision and were self-reported to be free from any history of neurological or psychiatric disorders. They participated after being fully informed of the experimental procedures and were paid. They were asked to watch 72 videos, with each video lasting about 2 s. After each video, they were asked to score the emotion reflected by the robot’s body movement (out of happiness, fear, anger, disgust, sadness, or surprise), followed by a short break. The order of the six emotional categories was random. A 7-point Likert scale was used, where 7 means strong and 1 means weak. The experiments were implemented in a well-lit and distraction-free room. Each participant spent an average of about 40 min. A scenario in which a student was watching video V41 is shown in Figure 3. All data obtained from the subjective evaluations were anonymized.

### 3.3. Results and Analysis

**Figure 4:**
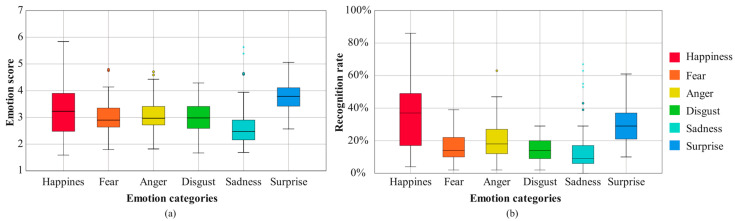
(**a**) The scores of 72 robot body movements in six emotional categories. (**b**) The recognition rates of 72 robot body movements in six emotional categories.

Each bodily expression of the robot shown in the video was scored under different emotional categories, where the scores indicate the intensity of emotion expressed by the robot’s body movement from the user’s view. The data were basically consistent with a normal distribution. The repeated-measures analysis of variance (ANOVA) was conducted to analyze these data. The data were divided into six groups, according to the scores in different emotional categories for all robot body movements. The Mauchly’s test of sphericity *p* < 0.05. In the tests for within-subject effects, F (5, 300) = 13.062, *p* < 0.001 partial *η*^2^ = 0.179. These results reveal that there exist significant differences in different emotional categories. In the pairwise comparisons, the scores for surprise showed a significant difference with the scores for other emotions, and the scores for happiness showed a significant difference from the scores for sadness. The mean values (M values) of scores for all robot body movements and for each emotion category are shown in Figure 4a. The overall trend shows relatively high scores for surprise and relatively low scores for sadness, while the scores were similar for happiness, fear, anger, and disgust. This indicates that, for all the designed robot body movements, expressions of surprise are easier to perceive than others, while expressions of sadness are harder to perceive than others.

The emotion recognition rate obtained in the subjective evaluation is defined as the ratio of the number of participants who score the highest for this emotion category to the total number of participants, and the data are shown in Figure 4b. The recognition rates for happiness were relatively better, but the overall trends were not high, especially for sadness, fear, and disgust. The Spearman correlation test was used to analyze the correlations between the recognition rates and the scores of robot body movements, and the results are shown in Table 1, where s represents the Spearman correlation coefficient. All *p*-values are smaller than 0.001. Happiness presented the strongest correlation, with s = 0.97. However, for surprise, the correlation value was only s = 0.569. Although there were relatively large differences in the correlations for different emotions, the results reveal that there are positive correlations with different intensities between the recognition rates and the scores of robot body movements in the six emotional categories. For example, the recognition of happiness was highly influenced by the intensity of emotion with happiness, while that of surprise was relatively less influenced by the intensity of surprise. For the robot’s emotional body movements, the impact of the intensities of emotions on the recognitions of negative emotions (including disgust, fear, sadness, and anger) were less than that for the recognition of happiness.

Hence, to help the users to recognize the emotions reflected by robot body movements easier, we built a dataset of robot emotional body movements by taking the recognition rate of more than 50% and the score of more than 3.5 as conditions. The robot body movements included in the dataset and the corresponding emotion recognition rate, M value, standard deviation (SD), etc., are detailed in Table 2. Then, 25 robot emotional body movements were selected, which better reflect the association between the robot’s body movements and the corresponding emotions. It should be noted that the eligible robot’s body movements after screening belonged to four emotional categories: happiness, anger, sadness, and surprise. We observed some differences between the initial design and the results. Some body movements of the Yanshee robot were designed to express a certain emotional state but were identified as another emotional state. For example, the body movement shown in V4 was designed to represent anger but was identified as happiness with a high recognition rate (86.27%). The body movement shown in V8 was designed for fear, but was also identified as happiness with a recognition rate of 74.51%. The body movement shown in V10 was designed for happiness but was identified as fear with a recognition rate of 37.25%.

## 4. Emotion Recognition Model Using BP Neural Network for Robot’s Body Movement

### 4.1. The Neural Network Topological Architecture of the Proposed Model

**Figure 5:**
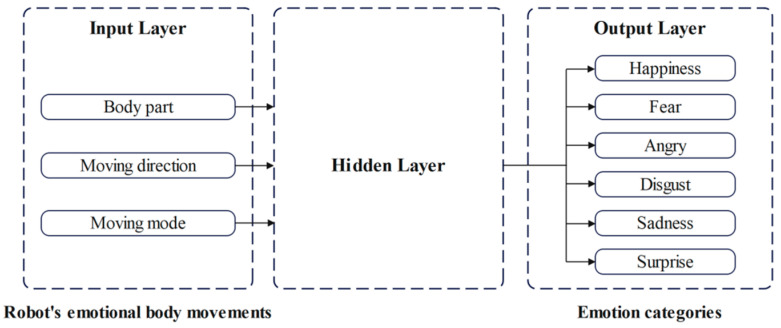
The neural network architecture of the proposed model.

The emotional recognition of robot body movements can be regarded as the mapping from the input layer to the output layer in a neural network, where the body movements of the robot and corresponding emotions can be used as the inputs and outputs of the neural network in the proposed model, respectively. In training the neural network, the proposed model gradually improves its emotion recognition ability through learning the mapping between characteristics of the robot’s body movements and the corresponding emotions. The architecture of the proposed model is given by Figure 5. The input layer consists of the information expressed by the robot’s body movements, including the moving body part, the direction of movement, and the moving mode. The output layer shows the emotions including happiness, fear, anger, disgust, sadness and surprise.

### 4.2. Encoding Rules

**Figure 6:**
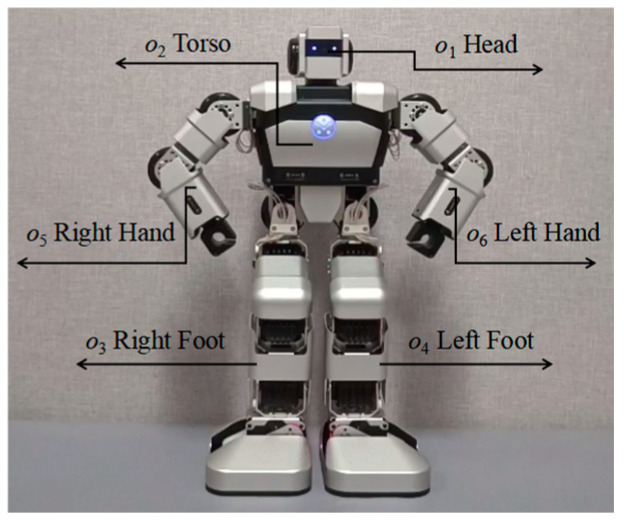
The body parts of Yanshee robot.

The robot emotional body movements in the dataset built in Section 3.3 had to be encoded to transform the movements into numerical forms, allowing them to be applied as the inputs in neural network training. When the body part, the direction of movement, or the moving mode is triggered, the corresponding node in the input layer of the neural network is set to 1, and the other nodes (being untriggered) are set to 0. Similarly, the node corresponding to the triggered emotion in the output layer is set to 1, while the other emotion nodes are set to 0. The motion state set for inputs and the emotion state set for outputs are illustrated by Table 3. The motion state set can be further divided into three subsets, reflecting the body part, moving direction, and moving mode. ***X_n_*** represents the input information in the motion state set, where *n* is the sequence number of the robot’s body movements. ***X_n_*** = {***O_n_***, ***D_n_***, ***M_n_***}, where ***O***, ***D,*** and ***M*** represent the body part, moving direction, and moving mode sets, respectively. The body parts of the Yanshee robot are shown in Figure 6. ***Y****_n_* = {*y*_1_, *y*_2_, *y*_3_, *y*_4_, *y*_5_, *y*_6_} represents the information in the emotion state set, where *y*_1_, *y*_2_, *y*_3_, *y*_4_, *y*_5_, and *y*_6_ represent happiness, fear, anger, disgust, sadness, and surprise, respectively. n∈[1, 72] is a positive integer. ***X_n_*** is the input coded sequence, which is used in the input layer of the neural network shown in Figure 5, while ***Y****_n_* is the corresponding output coded sequence.

**Figure 7:**
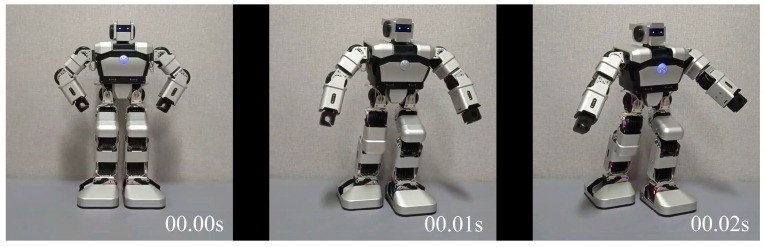
The body movements in V4 of Yanshee robot.

In the studies of Fu et al. [31], Yagi et al. [60], and Mahzoon et al. [61], the vertical features of robot body movements were considered to express emotions, and the studies of de Meijer [33] and De Silva et al. [34] referred to both vertical and lateral features, including the stretching and bowing movements of the body, to distinguish positive and negative emotions. Hence, when encoding the robot’s emotional body movements, the vertical and lateral movements are considered separately. Take the robot’s body movement in V4 as an example. In Figure 7, the body movements of the Yanshee robot over two seconds can be seen, which consist of two movements combined. One is from the vertical view, where the Yanshee robot’s head, torso, and left foot rotate to the front-left. The other is from the lateral view, where the robot’s hands are stretched forward simultaneously.

As the triggered and untriggered nodes are set to 1 and 0, respectively, considering Table 3, for the body parts, the nodes of the robot’s head, torso, left foot, left hand, and right hand are set to 1, while the node of the right foot is set to 0. For the moving direction, left and forward are 1 and the others are 0, and for the moving mode, rotation and extend are 1 and the others are 0. ***P***_4_, ***D***_4_, and ***M***_4_ are given by Equation (1). ***X***_4_ = {***P***_4_, ***D***_4_, ***M***_4_}.

(1) $$\left\{\begin{array}{c} P_{4}=\left\{1,1,0,1,1,1\}\right.\\ D_{4}=\left\{1,0,1,0,0,0,0,0\}\right.\\ M_{4}=\left\{1,0,0,1,0,0\}\right. \end{array}\right.$$

After all the movements are encoded, the corresponding emotional states are also considered. For example, for the robot’s body movement in V4, the corresponding emotion category is happiness, which means that ***Y***_4_ = {1, 0, 0, 0, 0, 0}.

### 4.3. The Proposed Emotional Body Movement Recognition Model Using BP Neural Network

**Figure 8:**
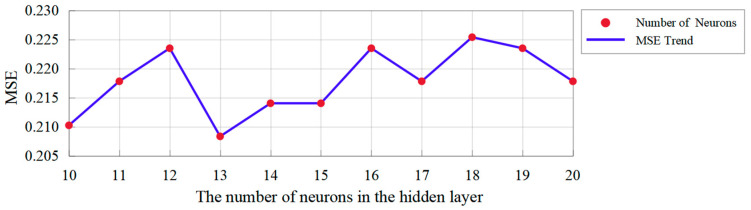
The MSEs with the number of hidden neurons.

As the robot’s emotional body movements designed in Section 3 are insufficient for recognizing the robot’s body movements with respect to the six emotion categories, we introduce our previous design reflecting twelve emotional body movements of the Yanshee robot, which has been verified to express the emotions of happiness, anger, sadness, fear, and disgust. In total, 37 emotional body movements of the Yanshee robot were employed, of which 28 were used to train the proposed neural network and 9 were used for the recognition experiments. The number of neurons in the hidden layer was determined using the Levenberg–Marquardt method, and the trend of the corresponding value of mean square error (MSE) is shown in Figure 8. Considering the minimum value of MSE, the corresponding number of neurons in the hidden layer was set to 13. Hence, the proposed emotion recognition model uses a BP neural network including an input layer with 20 nodes, a hidden layer with 13 nodes, and an output layer with 6 nodes. For convenience, the proposed emotion recognition model using a BP neural network is abbreviated as the EBMR-BP model.

The activation function, *logsig*(*x*), was used, as it can map the inputs into the range of (0, 1) to match the encoding for the robot’s body movements. The activations ***a*** and net activations ***z*** are given by Equations (2) and (3), respectively.

(2) $$a^{\left(l\right)}=logsig\left(W^{\left(l\right)}a^{\left(l-1\right)}+b^{\left(l\right)}\right),$$

(3) $$z^{\left(l\right)}=W^{\left(l\right)}logsig\left(z^{\left(l-1\right)}\right)+b^{\left(l\right)},$$

where *l* = 1, 2, which represents the number of neural network layers; $a^{\left(0\right)}=X$; and $a^{\left(2\right)}$ is the output of the neural network. The error of the *l*th neural layer, $\delta^{\left(l\right)}$, is shown in Equation (4):

(4) $$\delta^{\left(l\right)}={logsig}^{\cdot }\left(z^{\left(l\right)}\right)∙b^{\left(l\right)}\delta^{\left(l+1\right)}$$

**Figure 9:**
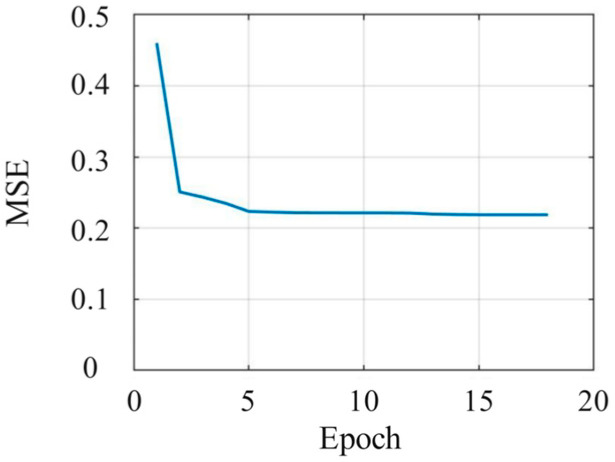
The MSEs of proposed BP model.

***W*** and ***b*** are the weight and bias matrices, respectively, which are updated using the LM method. The learning rate is set to 0.01, and the goal MSE is set to 0.00001. A total of 28 robot movements were randomly selected to train the proposed BP neural network, and some examples of the corresponding encoded inputs and outputs are illustrated in Table 4. The percentages of the training, validation, and test sets were 80%, 10%, and 10%, respectively. After training the EBMR-BP model, the MSE trend is shown in Figure 9. The minimum value of MSE was 0.219, which is not small enough.

## 5. The Optimization for Proposed Model Using PSO and RMSProp

The particle swarm optimization (PSO) method and the root mean square propagation (RMSProp) algorithm are used to optimize the proposed EBMR-BP model. The main aim of using PSO and RMSProp is to reduce the values of MSEs. The key steps are summarized as follows.

Step 1. Initialize the weights and biases of the proposed BP neural network, as well as the velocity and the position of particles to build the PSO-BP neural network. The positions of particles represent the weights and biases of the neural network, and the speed controls the corresponding state of movement in the search space. In the space of particles, the position matrix ***L*** and the speed matrix ***v*** of a particle are calculated using Equations (5) and (6), respectively.

(5) $$v_{s}^{t+1}=\omega v_{s}^{t}+c_{1}r_{1}\left(p_{s}^{t}-L_{s}^{t}\right)+c_{2}r_{2}\left(p_{g}^{t}-L_{s}^{t}\right),$$

(6) $$L_{s}^{t+1}=L_{s}^{t}+v_{s}^{t+1},$$

where *s* is the serial number of particles, *t* is the count of iterations, ***p****_s_* is the personal best (pBest), and ***p****_g_* is the global best (gBest). The learning factors *c*_1_ and *c*_2_ are set as 1.6 and 2, respectively; the random factors *r*_1_ and *r*_2_ are distributed between 0 and 1; and the inertia weight ω is set to be 0.6.

Step 2. Compute the fitness for particles in the space. The MSEs between the outputs of the proposed BP neural network and the target values are used as the fitness function. The speed of particles is limited by Equation (7), in order to ensure their convergence.

(7) $$v_{s}\left(t\right)=max\left(min\left(v_{s}\left(t\right),v_{max}\right),v_{min}\right),$$

where *v_max_* and *v_min_* are set to 0.5 and −0.5, respectively. Then, pBest and gBest are updated, and the position ***L*** and the speed ***v*** can be updated by Equations (2) and (3) until convergence or the maximum number of iterations is reached.

Step 3. The errors of each layer in the neural network are calculated, and the RMSProp algorithm is introduced when training the neural network. For the *l*-th layer, the gradients for ***W*** and ***b*** are given by Equations (8) and (9), respectively.

(8) $$\frac{\partial L}{\partial W^{\left(l\right)}}=\delta^{\left(l\right)}{\left(a^{\left(l-1\right)}\right)}^{T},$$

(9) $$\frac{\partial L}{\partial b^{\left(l\right)}}=\delta^{\left(l\right)},$$

where L represents the loss function. For the *t*-th iteration, the accumulations of gradient squares are given by Equation (10).

(10) $$G_{t}=\beta G_{t-1}+(1-\beta )g_{t}\cdot g_{t},$$

where the attenuation rate β = 0.9 and $g_{t}$ represents the gradients of parameters. Then, ***W*** and ***b*** can be updated until the stop condition is reached.

**Figure 10:**
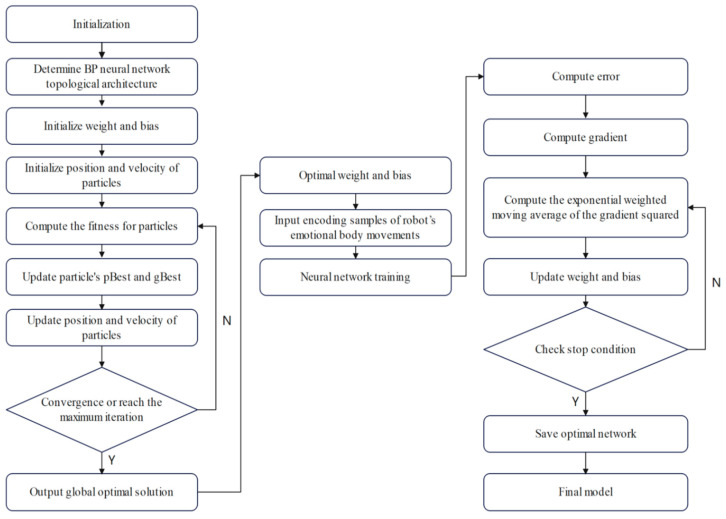
The main flow of PSO-BP-RMSProp model.

The above-mentioned process consists of training the PSO-BP neural network, then carrying out optimization using the RMSProp algorithm. For simplicity, it is abbreviated as the PSO-BP-RMSProp model. The main flow of this model is shown in Figure 10.

## 6. Experiments and Comparisons

**Figure 11:**
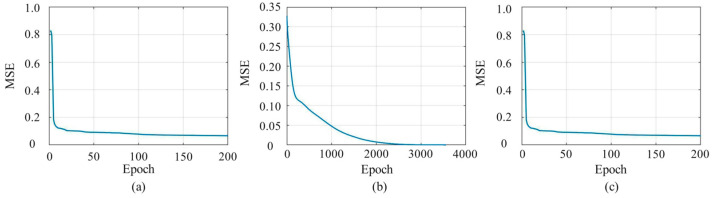
From (**a**–**c**), the MSEs of PSO-BP model, BP-RMSProp model and PSO-BP-RMSProp model with the epochs, respectively.

**Figure 12:**
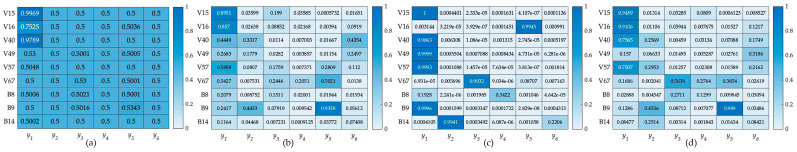
From (**a**–**d**), the data of emotion recognition obtained by proposed BP model, PSO-BP model, BP-RMSProp model and PSO-BP-RMSProp model, respectively.

Considering the EBMR-BP model proposed in Section 4.3 and steps 1 and 2 in Section 5, it is a process that involves optimizing the EBMR-BP model using the PSO method. When just considering the EBMR-BP model and step 3 in Section 5, the EBMR-BP model is optimized using the RMSProp algorithm. To simplify for discussion, these two are shortened to the PSO-BP model and BP-RMSProp model, respectively. Four models, including the EBMR-BP model, the PSO-BP model, the BP-RMSProp model, and the PSO-BP-RMSProp model, were used to recognize the emotions associated with the robot’s body movements. Nine robot emotional body movements were employed as the materials for recognition. The maximum number of iterations was 200 for the PSO-BP and PSO-BP-RMSProp models. For the BP-RMSProp model, the iterations stopped in the case of reaching the target error. The trends of MSEs for the PSO-BP, BP-RMSProp, and PSO-BP-RMSProp models with the epochs are shown in Figure 11a, Figure 11b, and Figure 11c, respectively. As shown in Figure 11b, the convergence ability of the BP-RMSProp model is not as good. The minimum MSEs for the PSO-BP, BP-RMSProp, and PSO-BP-RMSProp models were 0.0664, 9.9576 × 10^−6^, and 0.0664, respectively, and it was observed that the MSEs were smaller than that in Figure 9 in the case of convergence. This means that PSO and RMSProp are useful for reducing the MSE. The heatmaps of the data obtained with the EBMR-BP, PSO-BP, BP-RMSProp, and PSO-BP-RMSProp models are illustrated in Figure 12, where the horizontal axis represents different categories of emotion described in Section 4.2, and the vertical axis represents the serial number of the robot’s different body movements. The horizontal position of the maximum value for each row shows the corresponding emotional state of the robot’s body movement. It is obvious that, for the EBMR-BP model, the differences in the data were quite small, as shown in Figure 12a.

**Figure 13:**
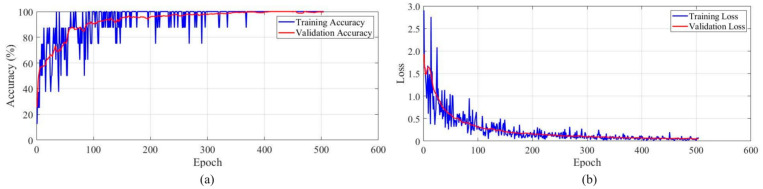
(**a**) The accuracy curve for each epoch of the CNN method. (**b**) The loss curve for each epoch of the CNN method.

A convolutional neural network (CNN) was used to compare the emotion recognition performance for the robot’s body movements. The architecture has a total of 12 layers. The videos used in Section 4.3 were sampled into images, where the sampling frequency was 30 kHz, and the input layer of the CNN takes these images as input. Then, a 2D convolutional layer with 16 filters and a kernel size of 3 × 3, a batch normalization layer, a ReLU layer, and a max pooling layer follow. The next four layers include another 2D convolutional layer with 32 filters and a kernel size of 3 × 3, a batch normalization layer, a ReLU layer, and a max pooling layer. These are followed by a dense layer with six nodes, which represents the six emotional categories shown in Section 4.2. The following Softmax activation layer and classification layer yield the output of the CNN. The accuracy and loss for each epoch of the CNN are shown in Figure 13a and Figure 13b, respectively. The comparisons between different methods for emotion recognition from robot body movements are given in Table 5, where Method I, II, and III represent the subjective emotion evaluation, emotion recognition using the proposed models, and emotion recognition using the CNN, respectively.

The subjective emotion recognition results were taken as the baseline. The numbers that could be recognized by the four proposed models were 6, 5, 4, and 8, respectively, while that of the CNN was 6. The emotion recognition rates of the EBMR-BP, PSO-BP, BP-RMSProp, PSO-BP-RMSProp, and CNN models were 66.67%, 55.56%, 44.44%, 88.89%, and 66.67%, respectively. This means that the proposed EBMR-BP model is effective and, when the PSO method and RMSProp algorithm are introduced separately to optimize the proposed BP model, they do not yield appropriate performances. However, when they are used in combination, the MSEs generated by the PSO-BP-RMSProp model are smaller than that generated by the EBMR-BP model, the convergence ability is the same as the PSO-BP model, and the corresponding emotion recognition rates are also improved. This indicates that optimization of the proposed model using PSO and RMSProp significantly improved its emotion recognition performance. The CNN method can be used for recognition, and its emotion recognition rate was the same as that of the EBMR-BP model. However, considering the results shown in Table 5, it is uncertain whether the number of robot body movements with other emotions (except for happiness) in the training dataset are sufficient for the CNN method. Extension of the dataset may lead to a better recognition rate for CNN, but the proposed PSO-BP-RMSProp model was able to achieve higher recognition rates with only the presently used data.

## 7. Conclusions

This study mainly focused on an emotional recognition method for a humanoid robot’s body movements. The Yanshee robot was employed, and its emotional body movements were designed by imitating the human body movements shown in the works of Zhang et al. and De Gelder et al. Subjective questionnaires were used, and the corresponding data were analyzed using statistical methods, including the repeated ANOVA and the Spearman correlation methods, in order to explore user perceptions and select appropriate robot emotional body movements. Then, a neural network topological architecture was built. The selected movements and corresponding emotions were used and encoded as its inputs and outputs, respectively. In this manner, an emotional body movement recognition model using a BP neural network for robot body movements—called the EBMR-BP model for convenience—was proposed. To optimize the proposed model, the PSO method and the RMSProp algorithm were introduced to reduce the MSE and improve the emotional body movement recognition rates. The PSO-BP-RMSProp model was developed, and the corresponding key steps were illustrated. The implementations of PSO-BP and BP-RMSProp were also induced on this basis. Experiments and comparisons of the emotional recognition of robot body movements were conducted using the EBMR-BP, PSO-BP, BP-RMSProp, and PSO-BP-RMSProp models, as well as a CNN model for comparison. The main contributions of this study are summarized as follows:

1. Positive correlations between the emotion recognition rates of robot body movements and the intensities of their emotional expressions were found. Moreover, the impacts on the emotion recognition rates differed for different emotions expressed by the robot’s body movements. The happiness recognition rates of robot body movements were greatly affected by the happiness expressions. Meanwhile, the influence of the robot’s negative emotion-related expressions on negative emotion recognition rates was lower than that of the happiness recognition expressions on the happiness recognition rates. Compared with other emotions, the expression of surprise showed the smallest relative effect on the recognition rates of surprise.
2. A dataset consisting of 25 robot emotional body movements and corresponding emotional states was designed, and it can be used as research material in future work focused on HRI.
3. The EBMR-BP model, which provides a feasible and effective approach to recognizing the emotions associated with a humanoid robot’s body movements, was proposed. The topological architecture, the encoding rule, and other aspects were described in detail. It allows for a mapping between a robot’s emotional body movements and human emotions to be successfully built using a computational model.
4. Optimization of the EBMR-BP model using a combination of the PSO method and the RMSProp algorithm was proposed to illustrate the feasible optimization path and verify the improvement in the emotion recognition ability of the robot’s emotional body movements. It also provides a feasible approach to achieve a relatively higher emotion recognition rate associated with body movements (88.89%) with a relatively small amount of data.

Overall, the findings of this study can provide beneficial references for future studies on self-adapting emotional recognition for robot body movements, as well as for the design of emotional interactions in the HRI context. Limitations are described from five aspects. First, the subjective results of robot body movements were obtained according to data obtained from young people. If the participants were from a wider range, the findings of this study may be more widely generalizable. Second, the participants of the subjective evaluation were Chinese. It is not certain whether the results would remain consistent in a different country or culture. Third, some subtle expressions of human body movements are hard to imitate when using the Yanshee robot due to its limited physical structure. This led to relatively large differences between the pre-designed robot body movements and the user perception results detailed in Section 3.2. Although appropriate body movements of the robot were selected in this work, more useable data may be obtained when using a robot with a higher number of DoFs. However, attaining such technical conditions was not possible in this study. Fourth, the effect of the moving speed of the robot’s joints on the subjective emotion evaluation was not considered, as it is uncertain how the variation in movement speed in different parts of robots could affect the evaluation. However, the speed may have some impacts, as some studies have shown that variation in robot velocity when moving forwards has impacts on human understanding [62], the positive and negative perceptions of observers [63], etc. Fifth, the Yanshee robot is a biped robot. As such, the encoding rules of the EBMR-BP and PSO-BP-RMSProp models in this study are in line with the characteristics of biped robots. In our view, their portability to other robot platforms is possible in theory. One reason is that our design was proposed through the imitation of human emotional body movements. Another reason is that robot body movements reflecting the same emotion show similar characteristics [33,34]. For a wheeled humanoid robot or other robot platforms with different physical characteristics, corresponding empirical studies are required. Our future work will focus on expanding the used dataset in order to develop models with better robustness and generalization ability, as well as further improving the emotion recognition rate of body movements.

## Figures and Tables

**Table 1:** The Spearman correlation coefficients between the recognition rates and the scores of robot’s body movements.

Correlation Coefficient	Happiness	Fear	Anger	Disgust	Sadness	Surprise
s	0.97	0.79	0.876	0.674	0.811	0.569

**Table 2:** The robot emotional body movements after screening.

Emotion	Video	Emotion Recognition Rate (%)	M Value	SD
Happiness	V4	86.27	5.16	1.63
	V39	86.27	5.84	1.60
	V8	74.51	5.24	1.89
	V28	72.55	5.10	1.73
	V21	70.59	5.12	1.76
	V20	68.63	5.00	2.38
	V3	64.71	4.12	1.70
	V22	62.75	4.43	1.93
	V71	60.78	4.06	1.95
	V44	60.78	4.06	1.99
	V31	58.82	4.08	1.79
	V55	58.82	4.39	2.30
	V15	58.82	4.61	2.01
	V40	54.90	4.51	2.12
	V57	50.98	4.02	1.97
	V67	50.98	3.98	2.20
	V16	50.98	4.10	2.08
Anger	V12	62.75	4.71	1.71
Sadness	V65	66.67	5.63	1.77
	V60	62.75	5.39	1.96
	V59	54.90	4.61	2.07
	V61	52.94	4.65	1.86
Surprise	V33	58.82	4.55	1.74
	V50	52.94	4.76	1.91
	V49	52.94	4.63	1.73

**Table 3:** The motion state set for inputs and the emotion state set for outputs.

The Motion State Set *X_n_*			The Emotion State Set *Y_n_*
Body Part	Moving Direction	Moving Mode	
Head	Left	Rotation	Happiness
Torso	Right	Swing	Fear
Right foot	Forward	Vertical	Anger
Left foot	Backward	Extend	Disgust
Right hand	Upward	Close up	Sadness
Left hand	Downward	Bend	Surprise
	Inward		
	Outward		

**Table 4:** Some examples of the encoded inputs and outputs for modeling proposed neural network.

Video	The Encoded Input	The Encoded Output
V4	{1, 1, 0, 1, 1, 1, 1, 0, 1, 0, 0, 0, 0, 0, 1, 0, 0, 1, 0, 0}	{1, 0, 0, 0, 0, 0}
V12	{0, 0, 1, 1, 1, 1, 0, 0, 1, 0, 0, 1, 0, 1, 1, 0, 0, 1, 0, 0}	{0, 0, 1, 0, 0, 0}
V33	{0, 0, 1, 1, 1, 1, 0, 0, 0, 0, 1, 0, 0, 1, 0, 0, 0, 1, 0, 1}	{0, 0, 0, 0, 0, 1}
V65	{0, 1, 0, 0, 1, 1, 0, 0, 0, 0, 1, 1, 0, 1, 0, 0, 0, 0, 1, 1}	{0, 0, 0, 0, 1, 0}

**Table 5:** Comparisons of emotion recognition results by different methods.

Video	Method I *	Method II *				Method III *
		EBMR-BP	PSO-BP	BP-RMSProp	PSO-BP-RMSProp	
V15	Happiness	Happiness	Happiness	Happiness	Happiness	Happiness
V16	Happiness	Happiness	Happiness	Sadness	Happiness	Happiness
V40	Happiness	Happiness	Happiness	Happiness	Happiness	Happiness
V49	Surprise	Happiness	Happiness	Happiness	Surprise	Happiness
V57	Happiness	Happiness	Happiness	Happiness	Happiness	Happiness
V67	Happiness	Anger	Sadness	Anger	Anger	Happiness
B8	Anger	Anger	Happiness	Disgust	Anger	Happiness
B9	Sadness	Sadness	Sadness	Happiness	Sadness	Happiness
B14	Fear	Happiness	Happiness	Fear	Fear	Happiness

* Methods I, II and III represent the subjective emotion evaluation, the emotion recognition by proposed models and the emotion recognition by CNN, respectively.

## Data Availability

The data presented in this study are available on request from the corresponding author. The data are not publicly available due to privacy.
